# Sensitive Periods of Moving on Mental Health and Academic Performance Among University Students

**DOI:** 10.3389/fpsyg.2019.01289

**Published:** 2019-06-13

**Authors:** Ming Li, Wen-Qiao Li, Liman Man Wai Li

**Affiliations:** ^1^Centre for Mental Health, Jishou University, Jishou, China; ^2^Department of Behavioral Science, Hokkaido University, Sapporo, Japan; ^3^Department of Psychology and Centre for Psychosocial Health, The Education University of Hong Kong, Tai Po, Hong Kong

**Keywords:** residential mobility, mental health, resilience, income, academic performance, sensitive periods

## Abstract

Moving within and across nations becomes a non-reversible increasing trend globally. The current research investigated the unique effect of residential mobility at different developmental stages (i.e., early childhood, late childhood, and adolescence) on university students’ mental health and academic performance. In addition, we investigated the role of two different types of coping resources, i.e., resilience and family income, in moderating the negative effect of residential mobility. The data from 3753 first-year university students revealed that: (1) residential mobility in late childhood and adolescence (but not in early childhood) predicted poorer mental health among university students; (2) high resilience and higher family income alleviated the association of residential mobility in adolescence and mental health status; and (3) residential mobility in adolescence (but not in early childhood and late childhood) was associated with poorer academic performance but this pattern was not moderated by resilience or family income. The theoretical implications and practical implications of these findings were discussed.

## Introduction

In recent years, high residential mobility within and across nations has gradually become an inevitable part in the contemporary world (Baldassare et al., 1979; Hilton and van Minnen, 2002; Ruback et al., 2004; Oishi et al., 2009). Researchers have been trying to uncover the effect of residential mobility on people’s lives across multiple domains, including attachment to community (Sampson, 1988), prosocial behaviors (Oishi et al., 2007b; Lun et al., 2012; Li et al., 2019, Li et al., unpublished), social relationships (e.g., Oishi et al., 2013; Li, 2017), and self-identity (Oishi, 2010). Among different domains, the research line that explores the influence of residential mobility on adjustment has been receiving a lot of attention. Specifically, whether residential mobility would have positive or negative consequences upon people’s mental health (Kessler and Magee, 1993; DeWit, 1998; Oishi and Graham, 2010; Boynton-Jarrett et al., 2013) is the key research question. In general, it was found that residential mobility has detrimental effects on people’s mental health (Kessler and Magee, 1993; DeWit, 1998; Oishi and Graham, 2010). For instance, prior work found that residential mobility in childhood was associated with an increased risk of developing a wide range of psychiatric disorders, including behavior-related disorders (e.g., suicidal behavior) and antisocial personality disorder, on reaching maturity (Jelleyman and Spencer, 2008; Green et al., 2010; Kessler et al., 2010; Brown et al., 2012; Boynton-Jarrett et al., 2013; Mok et al., 2016; Tseliou et al., 2016).

There were some limitations in previous studies. First, they mostly investigated the relationship between residential mobility and mental health without pinpointing the unique effect of residential mobility at different developmental stages. In other words, they ignored the possibility that moving experience may bring greater challenges at a specific developmental stage than in other development stages as suggested by some developmental theories (Erikson, 1963; Lin, 1983). In addition, mental health was primarily examined, thus it remains unknown whether the effect of residential mobility would extend to other aspects of functioning.

To address these concerns, the current research simultaneously examined the effect of residential mobility at different developmental stages, including early childhood (before age 6), late childhood (age 6–12), and adolescence (age 12–18), on university students’ mental health as well as their academic performance, an extensively used objective indicator of functioning at school (Glew et al., 2005; Keogh et al., 2006; Ramos-Sánchez and Nichols, 2007). Furthermore, we explored whether two types of coping resources, i.e., resilience (psychological capital) and family income (physical capital), would independently, or interactively play a moderating role in the effect of residential mobility on these two functioning aspects.

### Residential Mobility, Mental Health, and Academic Performance

A lot of theories contend a close relation between early experiences and current psychological experiences and outcomes. According to the classical Freud’s (1940) theory, people’s core personality traits are highly affected by the experience in childhood. Specifically, it highly depends on whether people have learned how to regulate and satisfy their desires in early developmental stages. The conditions, including fewer economic resources and higher level of residential mobility (Albrecht and Teachman, 2003; McGloin and Thomas, 2016) in childhood, are likely to undermine self-regulation, which can result in increased risks for mental health problems, and poor behavior outcomes during later developmental stages. This notion is in line with some empirical evidence showing that residential mobility impaired people’s self-control (Zhou et al., unpublished). In addition, based on the attachment theory, residential mobility, which causes disruption of peer relationship in childhood, is likely to link to interpersonal insecurity at later developmental stages (Bucci et al., 2015; Widom et al., 2018), which is a significant predictor of poor mental health and poor academic performance (Cordes et al., 2016; Langenkamp, 2016; Voight et al., 2017). Supporting the theories discussed above, empirical evidence exists in supporting the relation between residential mobility and mental health, and the relation between residential mobility and academic performance, which is outlined below.

#### Residential Mobility and Mental Health

Mental health is a significant determinant of individuals’ growth and well-being in the whole life span (Power and Hertzman, 1997; Bures, 2003; Ruddy et al., 2005; Jelleyman and Spencer, 2008; Oishi and Schimmack, 2010; Shiraga et al., 2013). Previous work shows that residential mobility has significant impacts on individuals’ mental health (Kessler and Magee, 1993; DeWit, 1998; Oishi and Graham, 2010), associating with more mental health problems (e.g., depression, schizophrenia, and anxiety) (Tunstall et al., 2014a,b). In general, frequent moving induces a series of stressors (e.g., family stress and household chaos), which can cumulate to bring detrimental impacts on mental health (Leventhal and Newman, 2010; Boynton-Jarrett et al., 2013).

#### Residential Mobility and Academic Performance

Some research has directly tested the relation between residential mobility and academic performance (e.g., Herbers et al., 2012; Voight et al., 2012; Schmitt et al., 2015), showing that moving experiences led to poorer academic performance among children, and adolescents (Herbers et al., 2012; Voight et al., 2012; Schmitt et al., 2015).

However, as stated previously, previous studies mostly tested the influence of total number of moving experiences and the short-term effect of residential mobility. It remains unclear whether the effect of residential mobility at different developmental stages would predict people’s mental health and academic performance in later developmental stages.

### The Effect of Residential Mobility in Different Developmental Stages

Individuals have different needs and developmental tasks to fulfill in each developmental stage (Erikson, 1963; Lin, 1983), suggesting that moving at certain developmental stages may bring greater challenges during the processes of achieving specific developmental tasks and goals. Thus it would be possible that residential mobility at different developmental stages would exert unique effect in different domains. To test this possibility, we followed the theory in the research of developmental psychology (Lin, 1983) to focus on three developmental stages: (a) early childhood – age before 6; (b) late childhood – age 6–12; (c) adolescence (age 12–18).

#### Sensitive Period of Moving on Mental Health

According to the personality development theory of Erikson (Erikson, 1963), individuals have to fulfill distinctive needs and developmental tasks to achieve better mental health in each developmental stage. Accumulated evidence converges to demonstrate that one of the most notable domains affected by residential mobility is social relationship (e.g., Sampson, 1988; Magdol and Bessel, 2003; Adam, 2004; Shklovski, 2007; Oishi, 2010; Sharkey and Sampson, 2010). Moving leads to destruction of one’s established social networks, which is a strong determinant of poor mental health (Greenblatt et al., 1982; Kawachi and Berkman, 2001). Therefore, changes in social relationships associated with residential mobility may be more detrimental to mental health in the developmental stage where the developmental task is highly related to social relationship.

Before age 6 (in early childhood), children mostly rely on their caregivers to overcome obstacles and develop a sense of security, therefore the quality of the relationship with the caregivers is the most important factor for their mental health (Lin, 1983), exerting short-term, and long-term influences (Bakeman and Brown, 1980; Crittenden, 1992; Mickelson et al., 1997). In contrast, previous research showed that children younger than age 6 were unlikely to have enduring peer relationships and group memberships (Ladd and Troop-Gordon, 2003). Thus, unless moving causes a separation with the caregivers, changes in social relationships caused by residential mobility at this stage were expected to have a minimal impact on mental health.

Since age 6 (in late childhood and adolescence), people begin to receive education with other people, which makes peer relationship to be a dominant factor of people’s psychological growth in this developmental stage (Armsden and Greenberg, 1987; Repper and Carter, 2011). As a result, people in late childhood and adolescence would become sensitive to changes in the living environments that could potentially affect their peer relationships (Hardy et al., 2002). Research on residential mobility consistently revealed that residential moves destroy extant social ties (Oishi, 2010; Oishi et al., 2013), thus it could be very challenging for people moving in late childhood or adolescence to maintain good relationships with their peers, which would in turn lead to poorer mental health. Moreover, the impacts upon peer relationships induced by residential mobility may serve to initiate mental health trajectories that can have a lasting effect on individuals, increasing their mental health vulnerabilities in later lives (Wadsworth, 1997).

Taken together, we expected that residential mobility after early childhood would have more notable detrimental effects on people’s mental health in later lives, whereas the effect of moving in early childhood on mental health would be rather minimal.

#### Sensitive Period of Moving on Academic Performance

As discussed previously, the personality development theory of Erikson (Erikson, 1963) proposed that the major developmental tasks for people in early and late childhood are to establish a secure relationship with their caregivers and peers, respectively. In contrast, the major developmental task in adolescence is related to “the self” (Erikson, 1963; Lin, 1983). That is to develop one’s self-identity through exploring the experience, need, emotion, ability, and goals that are related to “the self” (Kroger, 1989; Campbell and Lavallee, 1993). One way to develop one’s self-identity is through proving one’s ability. Therefore, self-identity achievement, which is crucial during adolescence, was found to be positively associated with academic performance (Sherman et al., 2013; Jelenic, 2014). Furthermore, the association between self-identity achievement and academic performance was also found among university students (Berzonsky and Kuk, 2005), indicating that the development of self-identity may have a long-term effect on academic performance.

Erikson (1968) proposed that sensing the change and deprivation of one’s environment is very likely to lead to role confusion among adolescents, leading to failure of accomplishing self-identity achievement. Hence, the changes in physical and social environments associated with residential mobility may be more detrimental to people’s academic performance when they move in adolescence. Inevitably, moving creates a lot of stressful situations, which causes tremendous pressure, and anxiety for individuals (Leventhal and Newman, 2010; Boynton-Jarrett et al., 2013; Tunstall et al., 2014a,b) and in turn, results in reduced self-control and delayed gratification (Oishi and Talhelm, 2012; Zhou et al., unpublished). These experiences associated with moving are likely to shape self-identity processes (Oishi, 2010; Eggleston and Oishi, 2013) that are more crucial in adolescence, which may in turn affect academic performance more notably (Culler and Holahan, 1980; Seipp, 1991; Bembenutty, 1999; Cassady and Johnson, 2002; Duckworth and Seligman, 2005; Hagger et al., 2010). Consistent with this notion, residential mobility was found to be correlated with poor identity achievement of adolescence (Oishi, 2010; Eggleston and Oishi, 2013) and in turn associated with poorer academic performance (Voight et al., 2012). Moreover, based on previous findings that self-identity development has long-term impacts on academic performance (Berzonsky and Kuk, 2005), the influence of moving experiences in adolescence on academic performance is likely to be extended to the university setting.

Therefore, we expected that moving frequency in adolescence would have a notable detrimental effect on academic performance among university students whereas the influence of moving frequency in early and late childhood would be minimal.

### The Moderating Role of Coping Resources

Some factors, including motivation of moving, the amount of time involved in moving (Scanlon and Devine, 2001), social support from significant others (Hendershott, 1989), and familial stability (Gilman et al., 2003), are identified to significantly moderate the outcomes of moving. To further examine the effect of residential mobility at different developmental stages on mental health and academic performance among university students, we also examined the moderating role of two types of coping resources: individuals’ resilience and their family income.

#### The Moderating Role of Resilience

Little research explored the moderating role of individual characteristics. In the present research, we tested the role of resilience, an important personality factor that predicts adjustments in a wide range of stressful situations (e.g., Rutter, 1985; Dumont and Provost, 1999; Bonanno, 2004; Campbell-Sills et al., 2006; Giallo and Gavidia-Payne, 2006; Troy and Mauss, 2011), on alleviating the impact of residential mobility. Resilience refers to the ability to recover from stress (Masten, 2007). People with higher resilience can adapt to stressful circumstances (Carver, 1998; Tusaie and Dyer, 2004). In general, people high in resilience tend to perceive more positive emotions and social support, which help to buffer stress (Tugade and Fredrickson, 2004; Friborg et al., 2005). In addition to its direct influence on adaptation, resilience can also alleviate the negative impact of some factors during adaptation. For instance, resilience reduced the incidence of schizophrenia in harsh living environments (Anthony, 1974).

Based on these findings, we speculated that high resilience would be likely to weaken the negative association between residential mobility and adjustment outcomes among university students. Specifically, we hypothesized that the influence of residential mobility in late childhood and adolescence on poorer mental health would be weaker among the university students with higher resilience. Similarly, we expected that the influence of residential mobility in adolescence on academic performance would be weaker among the university students with higher resilience.

#### The Moderating Role of Family Income

Another examined moderator is family income. Some previous studies suggested that the close association between exposure to childhood residential mobility and subsequent adverse outcomes could be restricted to households with lower family income, where a lot of psychosocial difficulties emerge (Webb et al., 2016). First, a weaker relation between residential mobility and adverse outcomes in households with higher family income may reflect aspirational residential movements for better employment, housing, and educational opportunities (Webb et al., 2016; Basolo and Yerena, 2017). In addition, family poverty has been identified to be a risk factor for a wide range of mental health and behavior issues among the youth (Letourneau et al., 2013; Reiss, 2013; Coley et al., 2018). In families with fewer economic resources, parents may not be able to provide sufficient emotional support to their children to cope with different challenges (Albrecht and Teachman, 2003), such as moving, which may eventually link to an increased risk for poor mental health and poor academic performance (Basolo and Yerena, 2017; Mollborn et al., 2018).

Based on previous findings, we expected that high family income would weaken the negative association between residential mobility and adjustment outcomes among university students. Specifically, we hypothesized that the influence of residential mobility in late childhood and adolescence on poorer mental health would be weaker among the university students with higher family income. Similarly, we expected that the influence of residential mobility in adolescence on academic performance would be weaker among the university students with higher family income.

### Overview of the Research

The primary aim of the current research was to investigate the unique effects of residential mobility at different developmental stages on different aspects of functioning in later lives. We recruited first-year students from a university in China to investigate how residential mobility at the three different developmental stages would affect university students’ mental health and academic performance. We hypothesized that: (a) residential mobility in late childhood and adolescence (but not early childhood) would predict poorer mental health among university students; (b) residential mobility in adolescence (but not in early childhood and late childhood) would predict poorer academic performance; and (c) higher resilience and higher family income would weaken the association of residential mobility with poor mental health and with poor academic performance. In addition, we explored whether resilience and family income would independently or interactively moderate the outcomes associated with residential mobility. Because both resilience and family income are important coping resources, we speculated that high resilience and high family income may jointly exert a stronger moderating effect.

## Materials and Methods

### Participants

Participants were 3753 first-year students (1436 male participants, 2317 female participants; *M*_age_ = 18.65, *SD* = 3.05) from a university in mid-West of China. Participants were majoring in a wide range of disciplines, including arts (53.2%), science (26.2%) and engineering (20.7%). There were 2611 Han Chinese participants, and 1142 participants with ethnic minority background, including Tujia Chinese (23.5%) and Miao Chinese (17.4%). All participants voluntarily participated in the current study, and they all gave their written informed consent before taking part in the study.

### Procedure and Measures

Participants completed the study through an online survey platform, i.e., Qualtrics. Using an online survey can protect participants’ privacy and make sure the test is done in a private setting with as little interference as possible from others. The following measures were used to test our research questions.

#### Residential Mobility

Modifying the items used in Oishi et al. (2007a)’s work, we asked participants to indicate their moving history at three different age periods. Participants reported the number of moving before age 6, between age 6 and 12, and after age 12.

#### Mental Health

To measure mental health, we used the Symptom Checklist 90 (SCL-90) scale (Derogatis et al., 1973), which includes 10 dimensions: somatization, obsessive-compulsive, interpersonal sensitivity, depression, anxiety, hostility, photic anxiety, paranoid ideation, psychoticism, and additional items (e.g., “Having headaches”; “Crying easily”; and “Having trouble falling asleep”). Participants indicated the extent to which they were affected by each of the problems described using a 5-point Likert-scale ranging from 1 (*not at all*) to 5 (*extremely*). The score of mental health was composited by summing up the scores of all items (α = 0.977, McDonald’s omega = 0.972), with higher scores indicating poorer mental health. This scale has been adopted in previous studies with Chinese participants (Li et al., 2016), and it was demonstrated to be reliable and valid among Chinese participants.

#### Academic Performance

We were able to obtain 3078 students’ scores of final exams at the first semester. Academic performance was computed by averaging the participants’ scores of all courses enrolled.

#### Family Income

Participants indicated their monthly family household income on a 7-point Likert scale, which ranged from 1 “*less than 1000 RMB*” to 7 “*more than 5000 RMB*.”

#### Resilience

Participants completed a resilience scale (Smith et al., 2008), which contains six items on a 5-point Likert-scale ranging from 1 (*strongly disagree*) to 5 (*strongly agree*). The sample items include, “I tend to bounce back quickly after hard times,” and “I have a hard time making it through stressful events” (reverse-scored item). An average score was computed (α = 0.743, McDonald’s omega = 0.730), with higher scores indicating higher level of resilience. Previous studies with Chinese participants have adopted this measure of resilience (Ye et al., 2017), which showed that this scale is reliable among Chinese participants.

### Statistical Analytic Plan

We analyzed the data using SPSS version 25 (I.B.M Corp, 2017) (and using R statistical software version 3.5.2 for McDonald’s omega; R Core Team, 2018). Four linear regression models were tested for each dependent variable (i.e., mental health and academic performance). All continuous variables were mean-cantered before computing interaction terms and entering into the analyses. In Model 1, in order to test the effect of residential mobility at different developmental stages, we regressed the dependent variable on residential mobility at the three developmental stages (early childhood – before age 6, late childhood – age 6–12, and adolescence – age over 12). In Model 2, we tested whether the results of Model 1 would remain similar with controlling for the effects of participants’ gender and age. In Model 3, we tested whether resilience and family income would exert moderating effects independently or interactively. Specifically, the dependent variable was regressed on the effect of residential mobility at each developmental stage, the effect of resilience, the effect of family income, the interaction of resilience and residential mobility at each developmental stage, the interaction of family income and residential mobility at each developmental stage, the interaction between family income and resilience, and the 3-way interaction of resilience, family income, and residential mobility at each developmental stage. In Model 4, we added age and gender as covariates based on Model 3 and tested whether the results would remain similar.

## Results

### The Effect of Residential Mobility at Different Developmental Stages on Mental Health

Table 1 presents the descriptive statistics of the key variables and their inter-correlations. Table 2 summarizes the results of all tested regression models. As indicated by the results of Model 1, participants’ residential mobility in late childhood, *b* = 2.193, *SE* = 1.017, *t* = 2.157, *p* = 0.031, 95% Confidence Interval (CI): [0.199, 4.187], and that in adolescence, *b* = 1.954, *SE* = 0.942, *t* = 2.075, *p* = 0.038, 95% CI: [0.108, 3.801], significantly predicted poorer mental health. In contrast, participants’ residential mobility in early childhood did not predict mental health, *b* = 0.464, *SE* = 1.029, *t* = 0.450, *p* = 0.652, 95% CI: [-1.554, 2.481]. Model 2 revealed that the results remained similar with controlling for the effect of gender and age. Residential mobility in late childhood, *b* = 1.885, *SE* = 1.020, *t* = 1.847, *p* = 0.065, 95% CI: [-0.116, 3.886], and residential mobility in adolescence, *b* = 2.273, *SE* = 0.942, *t* = 2.412, *p* = 0.016, 95% CI: [0.426, 4.121], were still associated with poorer mental health though the effect of residential mobility in late childhood was weakened. Furthermore, residential mobility in early childhood remained non-significant in predicting mental health, *b* = 0.559, *SE* = 1.031, *t* = 0.542, *p* = 0.588, 95% CI: [-1.463, 2.581]. In summary, the results indicated that residential moving experiences only after age 6 were associated with poor mental health among university students.

**Table 1:** The descriptive statistics of key variables and their inter-correlations.

	*M*	*SD*	1	2	3	4	5	6
1. MEC	0.370	0.732	–					
2. MLC	0.500	0.793	0.346**	–				
3. MA	0.470	0.800	0.164**	0.382**	–			
4. Resilience	3.350	0.658	0.002	–0.022	–0.020	–		
5. Family income	4.400	1.755	0.037*	0.054**	0.042*	0.051**	–	
6. Mental health	159.824	42.541	0.016	0.049**	0.053**	–0.402**	–0.079**	–
7. Academic performance	79.415	5.558	–0.028	–0.025	–0.051**	–0.028	–0.040*	0.003

*MEC, MLC, and MA represent residential mobility in early childhood, residential mobility in late childhood, and residential mobility in adolescence, respectively. ^∗^*p* < 0.05; ^∗∗^*p* < 0.01.*

**Table 2:** The results of the linear regression models tested for mental health and academic performance.

			Academic	
	Mental health		performance	
	*b*	*SE*	*b*	*SE*
**Model 1**				
Constant	159.12^∗∗∗^	0.704	79.421^∗∗∗^	0.1
MEC	0.464	1.029	–0.154	0.146
MLC	2.193^∗^	1.017	0.001	0.144
MA	1.954^∗^	0.942	–0.336^∗^	0.136
*F*	5.371^∗∗^		3.127^∗^	
Adjusted *R*^2^	0.004		0.002	
**Model 2**				
Constant	156.849^∗∗∗^	1.136	77.461^∗∗∗^	0.152
MEC	0.559	1.031	–0.206	0.141
MLC	1.885†	1.02	0.047	0.139
MA	2.273^∗^	0.942	–0.278^∗^	0.13
Gender	3.258^∗^	1.451	3.244^∗∗∗^	0.198
Age	–0.212	0.231	–0.468^∗∗∗^	0.098
*F*	4.401^∗∗^		63.622^∗∗∗^	
Adjusted *R*^2^	0.005		0.094	
**Model 3**				
Constant	159.04^∗∗∗^	0.643	79.434^∗∗∗^	0.101
MEC	0.588	0.945	–0.116	0.149
MLC	1.542^†^	0.932	0.008	0.145
MA	1.532^†^	0.86	–0.315^∗^	0.137
Resilience	–26.292^∗∗∗^	0.989	–0.197	0.153
Family income	–1.823^∗∗∗^	0.364	–0.123^∗^	0.058
Resilience^∗^MEC	1.486	1.558	0.077	0.243
Resilience^∗^MLC	–1.901	1.429	–0.157	0.223
Resilience^∗^MA	–2.42^∗^	1.211	–0.138	0.187
Family income^∗^MEC	0.012	0.528	0.11	0.085
Family income^∗^MLC	–0.223	0.547	–0.083	0.088
Family income^∗^MA	–1.13^∗^	0.482	–0.084	0.08
Resilience^∗^Family income	1.301^∗^	0.546	0.006	0.086
Resilience^∗^Family income^∗^MEC	0.403	0.901	–0.062	0.142
Resilience^∗^Family income^∗^MLC	1.101	0.844	–0.199	0.133
Resilience^∗^Family income^∗^MA	1.701^∗^	0.723	–0.081	0.115
*F*	55.025^∗∗∗^		1.845*	
Adjusted *R*^2^	0.179		0.004	
**Model 4**				
Constant	159.023^∗∗∗^	1.037	77.496^∗∗∗^	0.153
MEC	0.742	0.947	–0.166	0.144
MLC	1.269	0.934	0.053	0.14
MA	1.755^∗^	0.86	–0.254^†^	0.131
Resilience	–26.479^∗∗∗^	0.999	0.035	0.149
Family income	–1.803^∗∗∗^	0.365	–0.118^∗^	0.056
Resilience^∗^MEC	0.955	1.567	0.011	0.235
Resilience^∗^MLC	–1.804	1.442	–0.146	0.217
Resilience^∗^MA	–2.268†	1.216	–0.055	0.181
Family income^∗^MEC	0.031	0.528	0.094	0.082
Family income^∗^MLC	–0.315	0.548	–0.081	0.085
Family income^∗^MA	–1.1^∗^	0.481	–0.08	0.077
Resilience^∗^Family income	1.15^∗^	0.55	0.012	0.083
Resilience^∗^Family income^∗^MEC	0.465	0.902	–0.097	0.137
Resilience^∗^Family income^∗^MLC	0.908	0.853	–0.181	0.129
Resilience^∗^Family income^∗^MA	1.851^∗^	0.727	–0.045	0.111
Gender	–0.314	1.326	3.206^∗∗∗^	0.2
Age	–0.197	0.21	–0.494^∗∗∗^	0.098
*F*	48.422^∗∗∗^		19.549^∗∗∗^	
Adjusted *R*^2^	0.18		0.095	

MEC, MLC, and MA represent residential mobility in early childhood, residential mobility in late childhood, and residential mobility in adolescence, respectively. Unstandardized coefficients and standard errors are reported. ^†^*p*<0.1;*p<0.05;**p<0.01;***p<0.001.

#### The Moderating Role of Resilience and Family Income

As stated in Model 3, higher level of resilience, *b* = -26.292, *SE* = 0.989, *t* = -26.579, *p* < 0.001, 95% CI: [-28.231, -24.352], and higher level of family income, *b* = -1.823, *SE* = 0.364, *t* = -5.011, *p* < 0.001, 95%CI: [-2.536, -1.109], predicted better mental health. The interaction effect between resilience and family income was significant, *b* = 1.301, *SE* = 0.546, *t* = 2.381, *p* = 0.017, 95%CI: [0.230, 2.372].

Regarding the effects associated with residential mobility in early childhood, its main effect was non-significant, *b* = 0.588, *SE* = 0.945, *t* = 0.622, *p* = 0.534, 95% CI: [-1.265, 2.441]. The interaction effect of resilience and residential mobility in early childhood, *b* = 1.486, *SE* = 1.558, *t* = 0.953, *p* = 0.340, 95% CI: [-1.569, 4.541], and the interaction effect of family income and residential mobility in early childhood, *b* = 0.012, *SE* = 0.528, *t* = 0.022, *p* = 0.982, 95%CI: [-1.024, 1.047], were non-significant. The 3-way interaction effect of resilience, family income, and residential mobility in early childhood was also not significant, *b* = 0.403, *SE* = 0.901, *t* = 0.447, *p* = 0.655, 95%CI: [-1.364, 2.170].

Regarding the effects associated with residential mobility in late childhood, its main effect was marginally significant, *b* = 1.542, *SE* = 0.932, *t* = 1.654, *p* = 0.098, 95% CI: [-0.286, 3.370]. The interaction effect of resilience and residential mobility in late childhood, *b* = -1.901, *SE* = 1.429, *t* = -1.33, *p* = 0.183, 95%CI: [-4.703, 0.901], and the interaction effect of family income and residential mobility in late childhood, *b* = -0.223, *SE* = 0.547, *t* = -0.407, *p* = 0.684, 95%CI: [-1.296, 0.851], were not significant. The 3-way interaction effect of resilience, family income, and residential mobility in late childhood was also not significant, *b* = 1.101, *SE* = 0.844, *t* = 1.303, *p* = 0.192, 95%CI: [-0.555, 2.756].

Regarding the effects associated with residential mobility in adolescence, its main effect was marginally significant, *b* = 1.532, *SE* = 0.860, *t* = 1.781, *p* = 0.075, 95% CI: [-0.155, 3.218]. All two-way interactions associated with residential mobility in adolescence were significant: the interaction of resilience and residential mobility in adolescence: *b* = -2.420, *SE* = 1.211, *t* = -1.999, *p* = 0.046, 95% CI: [-4.793, -0.047]; the interaction effect of family income and residential mobility in adolescence: *b* = -1.130, *SE* = 0.482, *t* = -2.345, *p* = 0.019, 95%CI: [-2.075, -0.185]. Importantly, the interaction effect of resilience, family income, and residential mobility in adolescence was significant, *b* = 1.701, *SE* = 0.723, *t* = 2.353, *p* = 0.019, 95%CI: [0.284, 3.119].

**FIGURE 1:**
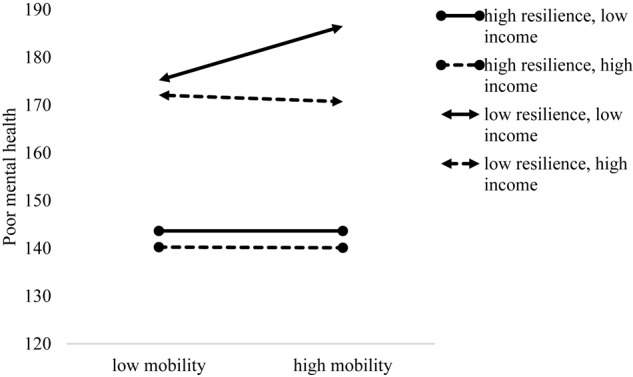
The interaction effect of resilience, family income, and residential mobility in adolescence in predicting university students’ mental health.

To unpack the 3-way interaction, simple-slope tests following the procedure suggested by Aiken and West (1991) were conducted. The results showed that residential mobility in adolescence was associated with poor mental health only when both resilience and family income were low (1*SD* below the mean), *b* = 7.073, *SE* = 1.679, *t* = 4.212, *p* < 0.001, 95%CI: [3.781, 10.365]; when both resilience and family were high (1*SD* above the mean), *b* = -0.083, *SE* = 1.600, *t* = -0.052, *p* = 0.959, 95%CI: [-3.221, 3.055], when resilience was high but family income was low, *b* = -0.003, *SE* = 1.726, *t* = -0.002, *p* = 0.999, 95% CI: [-3.386, 3.380], or when resilience was low but family income was high, *b* = -0.860, *SE* = 1.670, *t* = 0.515, *p* = 0.607, 95%CI: [-4.133, 2.414], residential mobility in adolescence was not associated with poor mental health (see Figure 1). The results remained similar when the effect of gender and age were controlled (see Table 1, Model 4).

### The Effect of Residential Mobility at Different Developmental Stages on Academic Performance

As stated in Model 1, the results showed that participants’ residential mobility in adolescence, *b* = -0.336, *SE* = 0.136, *t* = -2.470, *p* = 0.014, 95% CI: [-0.602, -0.069], significantly predicted poorer academic performance. However, participants’ residential mobility in early childhood, *b* = -0.154, *SE* = 0.146, *t* = -1.053, *p* = 0.293, 95% CI: [-0.440, 0133], and that in late childhood, *b* = 0.001, *SE* = 0.144, *t* = 0.006, *p* = 0.996, 95% CI: [-0.282, 0.283], did not predict poorer academic performance. The results remained similar when the effect of age and gender were controlled (Model 2): residential mobility in adolescence, *b* = -0.278, *SE* = 0.130, *t* = -2.129, *p* = 0.033, 95% CI: [-0.534, -0.022], residential mobility in early childhood, *b* = -0.206, *SE* = 0.141, *t* = -1.468, *p* = 0.142, 95%CI: [-0.482, 0.069], residential mobility in late childhood, *b* = 0.047, *SE* = 0.139, *t* = 0.336, *p* = 0.737, 95%CI: [-0.226, 0.319]. In summary, the results indicated that residential moving experiences only after age 12 was associated with poor academic performance among university students.

#### The Moderating Role of Resilience and Family Income

The results in Model 3 showed that the main effect of family income was significant with higher family income predicting lower academic performance, *b* = -0.123, *SE* = 0.058, *t* = -2.128, *p* = 0.033, 95%CI: [-0.236, -0.010]. In contrast, the main effect of resilience was not significant, *b* = -0.197, *SE* = 0.153, *t* = -1.285, *p* = 0.199, 95%CI: [-0.498, 0.104]. The interaction between resilience and family income was not significant, *b* = 0.006, *SE* = 0.086, *t* = 0.073, *p* = 0.942, 95%CI: [-0.162, 0.175].

Regarding the effects associated with residential mobility in early childhood, its main effect was not significant, *b* = -0.116, *SE* = 0.149, *t* = -0.776, *p* = 0.438, 95%CI = [-0.409, 0.177]. All interactions were not significant: the interaction effect between residential mobility in early childhood and resilience: *b* = 0.077, *SE* = 0.243, *t* = 0.315, *p* = 0.753, 95%CI: [-0.400, 0.553]; the interaction effect between residential mobility in early childhood and family income: *b* = 0.110, *SE* = 0.085, *t* = 1.291, *p* = 0.197, 95%CI: [-0.057, 0.276]; the 3-way interaction effect of resilience, family income, and residential mobility in early childhood: *b* = -0.062, *SE* = 0.142, *t* = -0.436, *p* = 0.663, 95%CI: [-0.340, 0.217].

Regarding the effects associated with residential mobility in late childhood, its main effect was not significant, *b* = 0.008, *SE* = 0.145, *t* = 0.056, *p* = 0.955, 95%CI = [-0.276, 0.293]. All interactions were not significant: the interaction between residential mobility in late childhood and resilience, *b* = -0.157, *SE* = 0.223, *t* = -0.702, *p* = 0.483, 95%CI: [-0.594, 0.281], the interaction between residential mobility in late childhood and family income, *b* = -0.083, *SE* = 0.088, *t* = -0.950, *p* = 0.342, 95%CI: [-0.256, 0.089], the 3-way interaction of resilience, family income, and residential mobility in late childhood, *b* = -0.199, *SE* = .133, *t* = -1.494, *p* = 0.135, 95%CI: [-0.460, 0.062].

Regarding the effects associated with residential mobility in adolescence, its main effect was significant, in which it significantly predicted poorer academic performance, *b* = -0.315, *SE* = 0.137, *t* = -2.305, *p* = 0.021, 95%CI: [-0.583, -0.047]. All interactions were not significant: the interaction between residential mobility in adolescence and resilience: *b* = -0.138, *SE* = 0.187, *t* = -0.738, *p* = 0.460, 95%CI: [-0.506, 0.229]; the interaction between residential mobility in adolescence and family income: *b* = -0.084, *SE* = 0.080, *t* = -1.052, *p* = 0.293, 95%CI: [-0.241, 0.073]; the 3-way interaction of resilience, family income and residential mobility in adolescence: *b* = -0.081, *SE* = 0.115, *t* = -0.707, *p* = 0.479, 95%CI: [-0.307, 0.144].

The results remained similar when the effect of age and gender were controlled (see Table 1, Model 4).

## Discussion

Previous research mostly investigated the relations between residential mobility and its outcomes such as mental health and academic performance without pinpointing the unique effects of residential mobility at different developmental stages. In the current research. First, we demonstrated that there was a sensitive period of moving on mental health. More moving experiences after age 6 was associated with poorer mental health of university students, whereas moving experiences before age 6 did not have notable impacts on mental health. Furthermore, resilience and family income moderated the negative effects of residential mobility in adolescence on mental health, in which high resilience or/and family income buffered the negative impact of residential mobility. Regarding academic performance, more moving experiences after age 12 predicted poorer academic performance of university students, and this pattern was not moderated by resilience or family income. Taken together, the findings suggested that residential mobility at different developmental stages had unique impacts on university students’ mental health and academic performance.

### Implications

#### Theoretical Implications

The current research had important implications for residential mobility research. On the one hand, some recent studies indicated that residential mobility can lead to positive consequences, including eliminating in-group favoritism in prosocial behaviors (Li et al., 2019), and promoting donations to a natural disaster (Li et al., unpublished). On the other hand, previous research generally suggests that residential mobility has negative consequences on individuals’ well-being or the overall well-being of society (Oishi, 2010; Oishi and Graham, 2010; Mok et al., 2016; Morris et al., 2018). In line with previous work, the current research found that residential mobility was associated with poor mental health and poor academic performance in the long run. Taking all existing evidence together, it suggests that whether the effect of residential mobility is positive or negative may be domain-specific. Future research should carefully explore what domains would be benefited or impaired by residential mobility.

Related to the above discussion, the current research provided a perspective to understand what domains would be impaired by residential mobility. We found a sensitive period of moving when we considered the relation of residential mobility at different developmental stages to mental health and academic performance among university students. Previous research primarily studied the overall effect of residential mobility on different domains (Leventhal and Newman, 2010; Boynton-Jarrett et al., 2013; Tunstall et al., 2014a,b; Glasheen et al., 2017). The current research highlighted the importance of the age at which people move, as moving experiences may bring greater challenges to the achievement of specific developmental goals suggested by some classic developmental theories (e.g., Erikson, 1963; Lin, 1983). Our findings showed that moving experiences after age 6 was associated with poorer mental health and moving experiences after 12 was associated with poorer academic performance among university students, which suggested that the sensitive period of moving could be different across domains. Similar to other research lines related to sensitive period (e.g., second language learning, and acculturation; Cheung et al., 2011; Blakemore and Mills, 2014), changes at certain time period in one’s life could bring stronger influence on some specific psychological processes. Future research should continue to explore how moving experiences at different developmental stages would shape people’s psychological processes in different domains.

#### Practical Implications

In addition to theoretical implications, the current research offered some important practical implications. Observing the negative effects of residential mobility on mental health and academic performance, parents should try to move as less as possible. However, residential mobility for better education or job opportunities has become an inevitable trend in modern society. The current research suggested that, instead of aborting the moving plan, parents can choose the right time to move, which could minimize the negative impact of residential mobility on mental health and academic performance in later lives.

In addition, the current research demonstrated the importance of two coping resources, in which having high residence or/and high income weakened the negative effect of residential mobility on mental health. These findings indicated that different types of capital, resilience as an important source of psychological capital and family income as an important source of physical capital, could buffer the negative effect of socio-ecological factors. First, consistent with previous studies (Albrecht and Teachman, 2003; Webb et al., 2016), family income is an important indicator of how much support or resources students can obtain to cope with difficulties associated with residential mobility (Rabe and Taylor, 2010), which can in turn promote better mental health. Despite its importance, it would be difficult to change students’ family income. More importantly, effective programs that can be implemented at school for promoting resilience, which was found to buffer the negative effect of residential mobility independently in the current research, are available. For instance, universities can implement group counseling service (Walsh, 2002), which may enhance students’ resilience and in turn help those with moving histories to attain better adjustments.

### Limitations and Future Directions

The current research had some limitations. First, the data in the current research were correlational, which did not provide causal evidence supporting the influence of residential mobility on mental health, and academic performance. Future studies should adopt a longitudinal design to observe how participants’ residential mobility at different developmental stages may potentially interact with their resilience and family income in affecting their experiences across different domains. Second, residential mobility may have different causes, which could be positive (e.g., seeking better employment, seeking better education environment), or negative (e.g., divorce). The moving experiences that differ in moving motives may have different effects on people’s psychological processes and behaviors (Rabe and Taylor, 2010). To fully understand the influence of residential mobility, we should carefully distinguish different types of moving and examine how different types of moving would affect people across domains in future research. Third, despite of a large sample size, we only conducted one study to test our research questions. Thus more studies are needed to check whether the findings could be replicated in other samples with different demographic or cultural characteristics. Fourth, we acknowledged that online studies could not strictly control the environmental characteristics as well as laboratory studies do. However, since the items in the measures for mental health are personally sensitive, using online surveys can ensure privacy, which can encourage participants to provide authentic response. Finally, we did not find a significant moderator that can alleviate the negative effects of moving on academic performance. Future research may consider investigating the role of other individual characteristics, such as self-esteem, in reducing the negative impact of residential mobility.

## Ethics Statement

This study was carried out with written informed consent from all participants. All participants gave written informed consent in accordance with the Declaration of Helsinki. The protocol was approved by the Departmental Ethics Review Board of Department of Psychology, Sun Yat-sen University.

## Author Contributions

ML and LL designed the study. ML collected data. ML and W-QL analyzed the data. ML, W-QL, and LL wrote the manuscript. LL supervised the project.

## Conflict of Interest Statement

The authors declare that the research was conducted in the absence of any commercial or financial relationships that could be construed as a potential conflict of interest.
